# Evaluation of Dexamethasone and Swimming Exercise as Complementary Interventions in a Rat Sciatic Nerve Injury Model

**DOI:** 10.3390/antiox14111382

**Published:** 2025-11-20

**Authors:** Meral Karakoç, Hayat Ayaz, Ferhat Çelik, Fırat Aşır

**Affiliations:** 1 Health Services Vocational School, Dicle University, Diyarbakir 21280, Turkey; meral.karakoc@dicle.edu.tr (M.K.); ferhat.celik@dicle.edu.tr (F.Ç.); 2 Department of Histology and Embryology, Medical School, Dicle University, Diyarbakir 21280, Turkey; ayazhayat44@gmail.com

**Keywords:** sciatic nerve injury, dexamethasone, swimming exercise, oxidative stress, GPX, MPO, myelin basic protein, peripheral nerve regeneration

## Abstract

Background: Peripheral nerve injuries frequently result in incomplete recovery despite advances in microsurgical repair. Both pharmacological and rehabilitative strategies have been investigated to enhance regeneration. Dexamethasone, a potent anti-inflammatory corticosteroid, and aerobic exercise, such as swimming, may promote repair through distinct but complementary mechanisms. Methods: A standardized rat sciatic nerve crush model was used to evaluate the effects of local dexamethasone administration (2 mg/kg/day, perineural for 10 days), swimming exercise (20 min/session, three times per week for 21 days), and their combination. Functional recovery was assessed by the Sciatic Functional Index (SFI), oxidative stress by MDA, GPX, and MPO assays, and structural recovery by histological, MBP immunohistochemical, and TEM analyses. Results: The injury group exhibited markedly elevated MDA and MPO levels and reduced GPX activity, indicating oxidative stress. Both dexamethasone and swimming exercise significantly improved these parameters, while the combination group showed values approaching controls (*p* < 0.001 for all comparisons vs. injury). Histological and immunohistochemical findings confirmed greater myelin preservation and higher MBP expression in treated groups, particularly in the combination group, whose g-ratio and myelin thickness were statistically indistinguishable from controls. SFI analysis revealed progressive motor improvement, with the combination therapy achieving near-normal function by day 28. Conclusions: This study demonstrates that dexamethasone and swimming exercise each contribute to peripheral nerve recovery and that their combined application provides additive benefits in terms of functional, biochemical, and structural regeneration. These results are limited to the specific dose and exercise regimen tested but support the potential of integrating anti-inflammatory pharmacotherapy with controlled physical exercise as a multimodal approach to enhance peripheral nerve repair.

## 1. Introduction

Peripheral nerve injuries (PNIs) represent a major clinical challenge, frequently resulting from trauma, surgery, or compression and often leading to long-term motor and sensory deficits [1]. The global incidence of PNIs has been estimated at approximately 11.2 cases per 100,000 person-years in England [2] and approximately 13.9 per 100,000 individuals in the Swedish population [3], with a global prevalence of about 2.8% among trauma cases [4]. Among experimental models, the sciatic nerve crush paradigm is most commonly conducted due to its well-defined anatomy, reproducible injury pattern, and the availability of standardized outcome measures [5]. Although peripheral nerves possess an inherent regenerative capacity, the process is typically slow and functionally incomplete, making the development of pharmacological and rehabilitative interventions that accelerate regeneration a key research priority [6].

Dexamethasone, a potent synthetic glucocorticoid, has been extensively studied for its ability to suppress inflammation, oxidative stress, and edema following nerve injury [7,8]. In rodent models, locally administered dexamethasone enhances axonal regeneration, reduces macrophage infiltration, and improves remyelination and functional recovery [9,10]. Local perineural delivery allows a higher concentration at the injury site while minimizing systemic adverse effects [11]. These anti-inflammatory and antioxidant actions collectively create a biochemical environment favorable for axonal survival and Schwann cell differentiation, both critical for successful regeneration [12,13].

In parallel, physical exercise has gained recognition as a non-pharmacological strategy to facilitate peripheral nerve repair [14]. Exercise has been shown to upregulate neurotrophic factors such as brain-derived neurotrophic factor (BDNF) [15] and growth-associated protein 43 (GAP43) [16], promoting axonal elongation and synaptic remodeling [17]. Moreover, exercise contributes to maintaining muscle mass and preventing denervation-induced atrophy, thereby improving the functional integration of regenerating nerves [18]. Among different exercise modalities, swimming offers unique advantages for rodent models: it is a low-impact, non-weight-bearing aerobic activity that reduces joint loading and postoperative stress while stimulating systemic circulation and neurotrophic signaling [19,20].

While the beneficial effects of dexamethasone and swimming exercise have been individually shown [21,22], few studies have examined their combined application in a unified experimental design. Given that dexamethasone primarily acts through anti-inflammatory and antioxidant mechanisms, whereas exercise enhances neuroplasticity through activity-dependent pathways, their additive or complementary effects may provide more comprehensive neuroprotection than either intervention alone [23,24]. Therefore, the aim of the present study was to investigate and compare the effects of dexamethasone, swimming exercise, and their combination on biochemical, histological, immunohistochemical, and ultrastructural recovery in a rat sciatic nerve crush model. We hypothesized that integrating pharmacological suppression of inflammation with activity-dependent neuroplasticity would yield enhanced functional and structural regeneration relative to monotherapy.

## 2. Materials and Methods

### 2.1. Animals and Ethical Approval

This study was conducted with the approval of the Local Animal Ethics Committee of Dicle University (Approval No: 2024/09, Date: 1 April 2024). All experimental procedures adhered to the NIH Guide for the Care and Use of Laboratory Animals and the ARRIVE guidelines [25]. A total of forty healthy adult male Wistar albino rats (250–300 g) were used and randomly allocated into five groups (*n* = 8 per group). Animals were housed under standard laboratory conditions (12 h light/dark cycle, temperature 23 ± 2 °C, relative humidity 50 ± 10%) with ad libitum access to food and water. Perioperative analgesia was provided with meloxicam (1 mg/kg, subcutaneous, once daily for 2 days) to minimize postoperative pain. Because the dosage and timing were identical across all experimental groups and the main biochemical and functional assessments were performed on day 28—beyond the drug’s pharmacological window—it is unlikely that meloxicam confounded the outcomes [26]. Anesthesia was induced via intramuscular ketamine (90 mg/kg) and xylazine (8 mg/kg).

### 2.2. Experimental Sciatic Nerve Crush Model

The sciatic nerve crush injury model was established as described by Zhou et al. [5] with minor modifications. Following aseptic preparation of the right gluteal region, the sciatic nerve was exposed through blunt dissection. A standardized compression injury was induced by applying a 2 mm wide hemostatic clamp for 60 s. The incision was then closed with absorbable sutures (Figure S1). To ensure sampling consistency, all tissues were collected from the same anatomical site: a 5 mm segment distal to the crush site.

Experimental Groups

- Control group: Sciatic nerve exposed but not crushed; saline (0.1 mL) injected perineurally for 10 days.
- Injury group: Nerve crushed; saline (0.1 mL) injected perineurally for 10 days.
- Injury + Swimming group: Nerve crushed, saline (0.1 mL) injected perineurally for 10 days, and then swimming protocol applied for 21 days.
- Injury + Dexamethasone group: Nerve crushed, only dexamethasone (2 mg/kg/day, local perineural injection at the injury site) administered for 10 days.
- Injury + Combination group: Nerve crushed, received dexamethasone (2 mg/kg/day, local perineural injection, 10 days) plus swimming exercise.

### 2.3. Swimming Exercise Protocol

The swimming protocol used in the present study—20 min per session, three sessions per week for 21 days—was selected based on prior reports showing that moderate-duration swimming enhances nerve regeneration without inducing excessive fatigue or stress responses [27,28]. All sessions were conducted during the light phase (10:00–14:00) under a controlled 12:12 light–dark cycle to minimize circadian variability. Pre-exercise acclimatization was provided to prevent acute stress, and water temperature was maintained at 28 ± 1 °C, which is optimal for minimizing corticosterone elevations while promoting voluntary movement [29]. These refinements ensured a moderate-intensity, physiologically tolerable regimen suitable for postoperative recovery.

**Figure 1:**
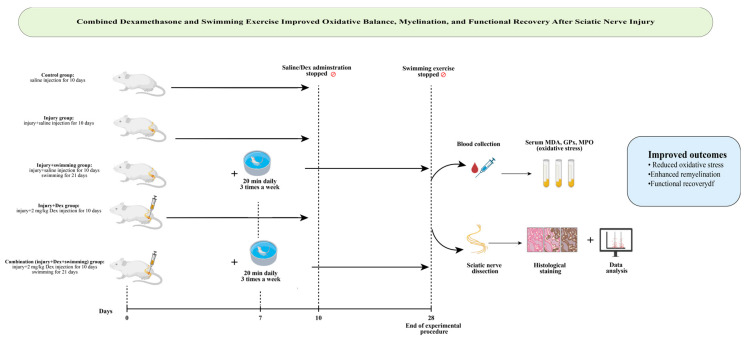
Graphical abstract illustrating study design and key findings: Dexamethasone and swimming exercise improved oxidative stress, remyelination, and functional recovery after sciatic nerve injury, with the combination therapy showing the most effective results (by https://www.biorender.com).

Swimming exercise was performed in a temperature-controlled tank (67 × 46.5 × 41 cm) filled with water maintained at 28 ± 1 °C [21]. Adaptation phase: One week prior to surgery, animals were acclimatized with short sessions (10, 20, and 30 min on consecutive days). Exercise phase: Starting on postoperative day 7, rats swam for 20 min per session, three sessions per week, for 3 weeks (total: 21 days). This protocol was chosen to avoid excessive physical stress and reduce the risk of wound complications, as longer or more frequent sessions may cause fatigue or infection in animals with postoperative muscle weakness. A graphical summary of the experimental design, including the main steps, timeline, and outcomes, is shown in Figure 1.

### 2.4. Functional Assessment of Sciatic Nerve

Motor recovery was assessed using the Sciatic Functional Index (SFI) on postoperative days 1, 7, 14, 21, and 28, as described by Varejao et al. [30]. Footprints were obtained from red-inked hind paws while animals traversed a paper-lined corridor. Measurements (paw length, toe spread, and intermediary toe spread) were recorded and SFI values calculated (Figure S2). All evaluations were performed by an investigator blinded to group allocation.

### 2.5. Biochemical Analysis

At the end of the experiment (day 28), blood was collected by cardiac puncture under anesthesia. Serum was separated by centrifugation and stored at −80 °C. Malondialdehyde (MDA) concentrations were measured with an ELISA kit (MyBioSource, San Diego, CA, USA; Cat. No: MBS268427) and expressed as nmol/mL. For glutathione peroxidase (GPX) analysis, approximately 300 mg of sciatic nerve tissue was homogenized in 500 µL of PBS (pH 7.2) using a glass homogenizer. For myeloperoxidase (MPO) analysis, 100 mg of tissue was homogenized in 1 mL of 1× PBS and incubated at −20 °C overnight. The homogenates prepared for both analyses were subjected to two freeze–thaw cycles and then centrifuged at either 1500× *g* for 15 min (GPX) or 5000× *g* for 5 min (MPO). The resulting supernatants were used immediately for analysis or aliquoted and stored at −80 °C. GPX levels were measured using a commercial ELISA kit (MyBioSource, USA; Cat. No: MBS744364), and MPO levels were measured using an ELISA kit (MyBioSource, USA; Cat. No: MBS704859), both in accordance with the manufacturer’s instructions. Results for both enzymes were reported as ng/g tissue.

### 2.6. Histological and Immunohistochemical Analyses

Sciatic nerves were fixed in 10% buffered formalin, processed routinely, and embedded in paraffin. Sections (5 µm) were stained with hematoxylin–eosin (H&E) for general histology and toluidine blue for myelin morphology. For immunohistochemistry, sections were incubated with anti-Myelin Basic Protein (MBP, catalog no: sc-271524, Santa Cruz Biotechnology, Dallas, TX, USA) following antigen retrieval and blocking steps. A streptavidin–peroxidase system with DAB chromogen was used for visualization. MBP-positive fibers were evaluated by a blinded observer [31,32]. For MBP quantification, five randomly selected high-power fields (400×) were analyzed per nerve section. Regions of interest (ROI) were defined to include the endoneurial area, excluding large blood vessels. The percentage of MBP-positive fibers was determined using Qupath software (version 0.5.0; University of Edinburgh, Edinburgh, UK, access date: 20 June 2025) with a uniform threshold across all images. Two independent blinded pathologists performed the analysis, and inter-rater reliability was verified (ICC = 0.93).

### 2.7. Ultrastructural Analysis

Separate sciatic nerve samples were fixed in 2.5% glutaraldehyde and processed for transmission electron microscopy (JEOL JEM-1010). Ultrathin sections (70 nm) were stained with uranyl acetate and lead citrate and then examined for axonal integrity, myelin thickness, and g-ratio.

### 2.8. Randomization and Blinding

Group allocation was determined using a random-number generator. All functional (SFI), biochemical, histological, and immunohistochemical analyses were performed by observers blinded to group identity.

### 2.9. In Silico Analysis

To elucidate the molecular mechanisms underlying dexamethasone-mediated neuroprotection, potential protein targets of dexamethasone and their associated Kyoto Encyclopedia of Genes and Genomes (KEGG) signaling pathways were analyzed using bioinformatics tools. Protein–protein interactors were identified and visualized in Cytoscape software (version 3.10.2) with a confidence cutoff of 0.4. Subsequently, the identified protein interactors were subjected to KEGG pathway enrichment analysis using the ShinyGO (version 0.85) [33], and the top 10 most significantly enriched pathways were determined based on fold enrichment (false discovery rate (FDR) cutoff: 0.05) [32,34].

### 2.10. Molecular Docking Analysis

Molecular docking simulations were performed to evaluate the binding affinity between dexamethasone and the key target proteins identified from the bioinformatic analysis (NF-κB, HIF-1α, CASP3, NOS2, TNF, PARP1, LEPR, and NR3C1), as well as the structural myelin protein Myelin Basic Protein (MBP). The three-dimensional structures of these proteins were retrieved from the Protein Data Bank (PDB; https://www.rcsb.org/). The 3D structure of dexamethasone (PubChem CID: 5743) was obtained in .sdf format from the PubChem database and converted to .pdbqt format using Open Babel (version 3.1.1). All protein and ligand files were prepared for docking by removing water molecules, adding polar hydrogens, and assigning Kollman charges using AutoDock Tools (version 1.5.7). Docking was carried out using AutoDock Vina (version 1.2.3) with an exhaustiveness parameter of 8. The grid box was defined to cover the active or ligand-binding region of each target protein, and coordinates were optimized to encompass all potential binding sites. For MBP docking, a grid box of 20 × 20 × 20 Å centered at the binding pocket identified through cavity prediction was applied. The best docking pose was selected based on the lowest binding energy (kcal/mol) and the number of hydrogen bond interactions. Visualization and interaction analysis were performed using Discovery Studio Visualizer (BIOVIA, San Diego, CA, USA) and PyMOL (version 2.5). The 2D ligand–protein interaction diagrams were generated using LigPlot+ software.

### 2.11. Statistical Analysis

Data were analyzed using IBM SPSS Statistics version 25 (IBM Corp., Armonk, NY, USA). Normality of distribution was verified with the Shapiro–Wilk test. Sciatic Functional Index (SFI): Because SFI was measured repeatedly in the same animals at five time points (days 1, 7, 14, 21, and 28) across five experimental groups, a two-way repeated measures ANOVA was used to assess the effects of group (between-subjects factor) and time (within-subjects factor), as well as their interaction (group × time). Tukey’s post hoc test was applied for pairwise comparisons. MDA, MBP immunoreactivity, histological scores, and TEM parameters (g-ratio): These variables were measured once at the experimental endpoint (day 28) and compared across groups using one-way ANOVA followed by Tukey’s post hoc test. For all analyses, effect sizes (η^2^) and F-values were reported where applicable. Multiple comparisons were corrected using the Benjamini–Hochberg false discovery rate (FDR) method to minimize type I error. Results are presented as mean ± standard deviation (SD), and statistical significance was set at *p* < 0.05.

## 3. Results

### 3.1. Dexamethasone and Swimming Markedly Reduce Oxidative Stress Following Sciatic Nerve Injury

The injury group showed the highest MDA and MPO levels and the lowest GPx activity, confirming the presence of severe oxidative stress following sciatic nerve crush on day 28, (Table 1). Both Dexamethasone and Swimming Exercise significantly attenuated oxidative stress, while the combination therapy provided the most pronounced improvement, closely approaching control values.

### 3.2. Combined Therapy Restores Motor Function to Near-Normal Levels by Day 28

SFI values demonstrated progressive functional recovery across all treatment groups (Table 2), while the injury group showed persistent deficits. The Combination group exhibited significantly greater improvement compared with Swimming or Dexamethasone alone starting at day 7 and maintained superior recovery through day 28 (two-way repeated measures ANOVA, group × time interaction *p* < 0.001; Tukey post hoc, all *p* < 0.001 after day 7). For example, on day 28, the Combination group (−7.25 ± 0.83) had near-normal values, significantly better than Dexamethasone (−20.75 ± 1.23) and Swimming (−27.36 ± 0.99).

### 3.3. Combined Treatment Preserves Axonal Integrity and Minimizes Structural Degeneration

**Figure 2:**
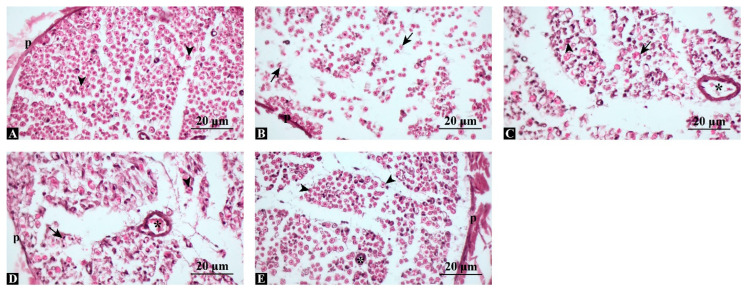
Representative hematoxylin and eosin (H&E)-stained sections of sciatic nerves. (**A**) Control group: compact, regularly organized axons with intact perineurium. (**B**) Injury group: marked axonal degeneration and myelin disruption. (**C**) Injury + Swimming: partial preservation of axonal continuity. (**D**) Injury + Dexamethasone: improved axonal alignment and myelin integrity compared with injury. (**E**) Injury + Combination: near-normal histological structure, closely resembling control. p: perineurium, arrowhead: regular axon, arrow: degenerated axon, asterisk: blood vessel, Scale bars: 20 μm.

H&E staining of cross-sections revealed normal nerve architecture in controls, with compact axons surrounded by intact perineurium. In the injury group, marked axonal degeneration and myelin sheath disorganization were evident. Both swimming and dexamethasone groups showed partial preservation of axonal continuity, though signs of degeneration remained. Notably, the combination group exhibited near-complete structural preservation resembling the control group (Figure 2).

**Figure 3:**
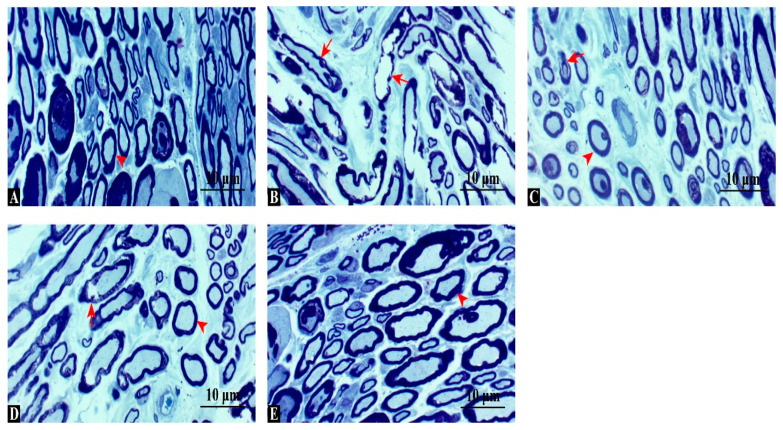
Toluidine blue-stained cross-sections of sciatic nerves. (**A**) Control group: dense, uniformly thick myelinated fibers. (**B**) Injury group: disorganized axons, myelin fragmentation, and vacuolization. (**C**) Injury + Swimming: partial improvement with more compact fibers. (**D**) Injury + Dexamethasone: better preservation of myelin structure and fewer degenerating fibers. (**E**) Injury + Combination: densely packed, regularly organized fibers, comparable to controls. Arrowhead: regular axon, arrow: degenerated axon, Scale bars: 10 μm.

Toluidine blue staining (Figure 3) revealed normal, dense myelinated axons in the control group, whereas the injury group showed marked myelin disintegration and axonal shrinkage. Swimming and dexamethasone treatments each provided partial structural protection, while the combined therapy preserved myelin integrity most effectively, closely resembling controls. These results indicate that combined treatment offers superior structural protection and promotes myelin regeneration compared with single interventions. Quantitative analysis of toluidine blue-stained sections confirmed these observations. The density of myelinated fibers (fibers/field, 400× magnification) was significantly reduced in the injury group (mean ± SD: 182 ± 16) compared with the control (415 ± 22, *p* < 0.001). Both swimming (276 ± 18) and dexamethasone (324 ± 20) improved fiber density, while the combination group showed the highest values (387 ± 21), approaching control levels (ANOVA F(4,35) = 162.8, *p* < 0.001, η^2^ = 0.95).

### 3.4. MBP Expression Is Significantly Enhanced, Indicating Improved Remyelination

**Figure 4:**
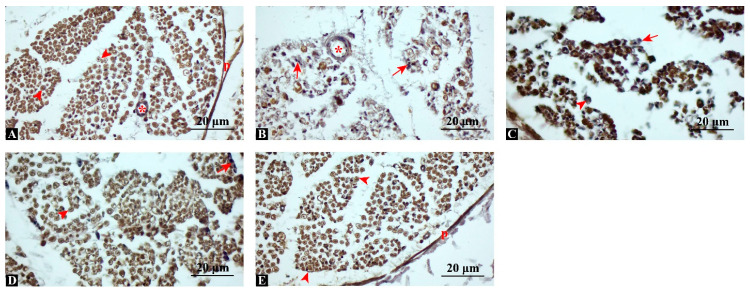
Immunohistochemical staining for myelin basic protein (MBP). (**A**) Control: strong, diffuse MBP positivity indicating intact myelin. (**B**) Injury: fragmented and reduced MBP staining, consistent with demyelination. (**C**) Injury + Swimming: moderate MBP positivity, with partially preserved fibers. (**D**) Injury + Dexamethasone: more prominent and continuous MBP expression. (**E**) Injury + Combination: robust MBP immunoreactivity, comparable to control group. p: perineurium; arrowhead: MBP-positive axon; arrow: MBP-negative axon, asterisk: blood vessel, Scale bars: 20 μm.

MBP immunostaining revealed strong, continuous positivity in control nerves, while the injury group showed fragmented and markedly reduced expression (Figure 4). Semi-quantitative scoring (percentage of MBP-positive fibers per high-power field, 400×) demonstrated that MBP expression was significantly preserved in treated groups. The swimming group demonstrated moderate MBP positivity (52.3 ± 4.8%), dexamethasone resulted in a higher level of positivity (68.9 ± 5.4%), and the combination therapy nearly restored expression to control values (88.1 ± 6.2% vs. control 92.5 ± 5.1%). Statistical analysis confirmed significant group differences (ANOVA F(4,35) = 104.5, *p* < 0.001, η^2^ = 0.92; post hoc *p* < 0.001 for Combination vs. Injury, Swimming, and Dexamethasone). These findings suggest that the combined intervention most effectively restored myelin integrity.

### 3.5. TEM Confirms Thicker Myelin and Lower G-Ratio Values with Combination Therapy

**Figure 5:**
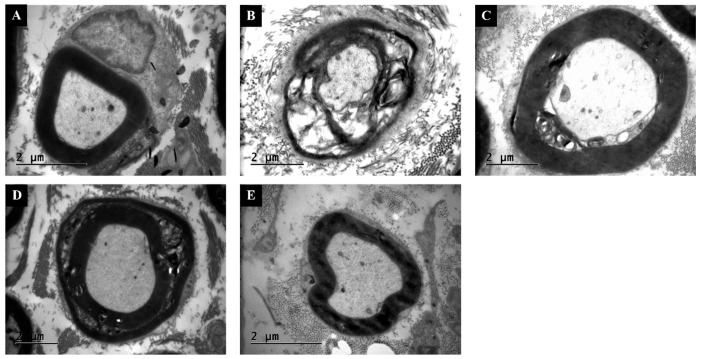
Transmission electron microscopy (TEM) images of sciatic nerves. (**A**) Control: normal axonal profiles with compact myelin lamellae. (**B**) Injury: extensive lamellar separation, axonal shrinkage, and vacuolization. (**C**) Injury + Swimming: partial recovery, with thicker myelin sheaths and improved organization. (**D**) Injury + Dexamethasone: reduced demyelination and more regular axonal profiles. (**E**) Injury + Combination: preserved ultrastructure with continuous myelin layers and uniformly shaped axons. Scale bars = 2 μm.

TEM analysis showed compact, regularly laminated myelin sheaths in the Control group (Figure 5). The Injury group displayed extensive lamellar disruption, vacuolization, and axonal atrophy. Swimming and Dexamethasone treatments partially reduced demyelination, while the Combination group exhibited nearly intact myelin layers and large, uniformly shaped axons. Quantitative ultrastructural analysis supported these observations. Mean g-ratio values (axon diameter/fiber diameter) were significantly higher (indicative of thinner myelin) in the Injury group (0.82 ± 0.04) than in Control (0.65 ± 0.03, *p* < 0.001). Both Swimming (0.74 ± 0.05) and Dexamethasone (0.71 ± 0.04) improved g-ratio, while the Combination group (0.67 ± 0.03) was statistically indistinguishable from Control (*p* > 0.05). Myelin thickness measurements followed a similar trend, with Combination showing the greatest preservation (ANOVA F(4,35) = 189.6, *p* < 0.001, η^2^ = 0.96).

### 3.6. Bioinformatic Analysis Links Dexamethasone Targets to NF-κB, HIF-1, and Oxidative Stress Pathways

**Figure 6:**
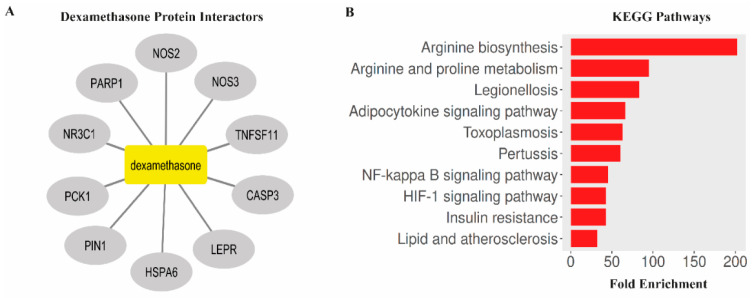
Bioinformatics analysis of dexamethasone-associated protein interactors and enriched KEGG pathways. (**A**) Protein–protein interaction network of dexamethasone targets generated using Cytoscape software. (**B**) KEGG pathway enrichment analysis showing the top 10 significantly enriched pathways.

Bioinformatics analysis identified several key protein interactors associated with dexamethasone, including caspase-3 (CASP3), nuclear receptor subfamily 3 group C member 1 (NR3C1), peptidyl-prolyl cis-trans isomerase NIMA-interacting 1 (PIN1), nitric oxide synthase 3 (NOS3), tumor necrosis factor ligand superfamily member 11 (TNFSF11), phosphoenolpyruvate carboxykinase 1 (PCK1), leptin receptor (LEPR), nitric oxide synthase 2 (NOS2), poly(ADP-ribose) polymerase 1 (PARP1), and heat shock protein family A member 6 (HSPA6). KEGG pathway enrichment analysis (FDR < 0.05) revealed that these proteins were significantly associated with multiple biological pathways, including arginine biosynthesis, arginine and proline metabolism, Legionellosis, adipocytokine signaling pathway, toxoplasmosis, pertussis, NF-κB signaling pathway, HIF-1 signaling pathway, insulin resistance, and lipid and atherosclerosis pathways. Collectively, these findings suggest that dexamethasone may exert its neuroprotective effects through modulation of metabolic, inflammatory, and oxidative stress-related signaling mechanisms (Figure 6).

### 3.7. Dexamethasone Shows Strong Binding Affinity to NF-κB and CASP3 in Silico

**Figure 7:**
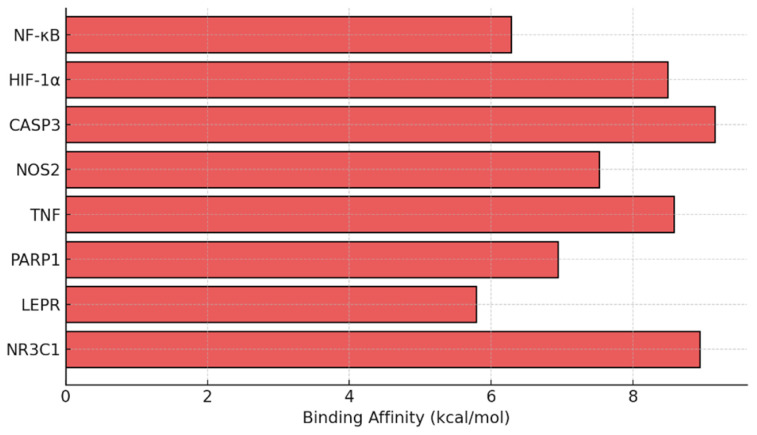
Predicted docking affinity of dexamethasone with target proteins based on bioinformatics analysis of dexamethasone-associated protein interactors and enriched KEGG pathway.

Molecular docking simulations were performed to predict the binding affinity of dexamethasone with key target proteins identified in the bioinformatic network (NF-κB, HIF-1α, CASP3, NOS2, TNF, PARP1, LEPR, and NR3C1). The docking results revealed strong interactions, particularly with NF-κB (≥−6 kcal/mol) and CASP3 (≥−6 kcal/mol), indicating the potential modulation of inflammatory and apoptotic signaling cascades by dexamethasone. These in silico findings complement the experimental data and provide molecular-level evidence supporting the observed neuroprotective outcomes (Figure 7).

### 3.8. Dexamethasone Interacts with MBP, Suggesting Structural Myelin Stabilization

**Figure 8:**
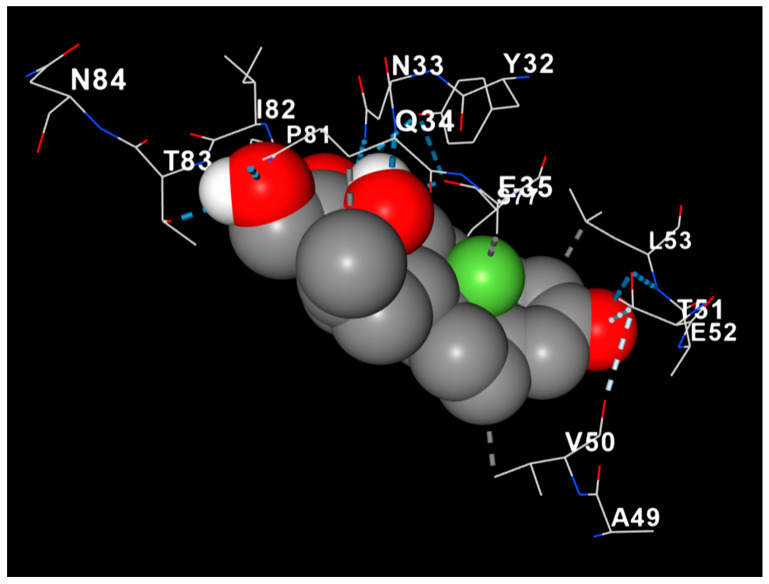
Molecular docking model of dexamethasone (gray spheres) within the binding pocket of Myelin Basic Protein (MBP). The protein backbone is displayed as a cartoon gradient (red to blue), and key interacting residues are labeled. Hydrogen bonds are indicated by dashed lines. The docking pose demonstrates a stable interaction (−8.5 kcal/mol) involving residues Val50, Glu52, Leu53, and Ala49, suggesting potential conformational stabilization of MBP.

Docking analysis revealed a stable interaction between dexamethasone and Myelin Basic Protein (MBP), with the ligand occupying a hydrophobic pocket formed mainly by residues Val50, Glu52, Leu53, and Ala49 (Figure 8). The complex was stabilized through both hydrophobic contacts and hydrogen bonding, with an estimated binding energy of approximately −8.5 kcal/mol. The steroid nucleus of dexamethasone was positioned parallel to the β-sheet surface of MBP, suggesting potential conformational stabilization. These findings imply that dexamethasone may contribute to myelin protection not only through anti-inflammatory signaling but also via direct molecular interaction with structural myelin proteins.

## 4. Discussion

The present study demonstrates that both dexamethasone and swimming exercise independently promoted functional, biochemical, and structural recovery following sciatic nerve crush injury in rats and that their combined application provided the most pronounced neuroprotective effects. These findings indicate that integrating pharmacological suppression of inflammation with activity-dependent neuroplasticity can yield additive benefits in peripheral nerve regeneration. Dexamethasone exerted its effects mainly by suppressing inflammation and oxidative stress [35]. This observation aligns with earlier studies reporting that local dexamethasone administration reduces macrophage infiltration, preserves axonal morphology, and accelerates Wallerian degeneration resolution [36,37]. In our experiment, the decrease in MDA and MPO levels, together with the restoration of GPX activity, indicates that dexamethasone effectively attenuated lipid peroxidation and enhanced the endogenous antioxidant defense system. Conversely, swimming exercise promoted recovery through activity-dependent mechanisms. Previous research has shown that aerobic exercise enhances expression of BDNF, IGF-1, and GAP-43, which support axonal outgrowth and remyelination [38,39]. Our histological and immunohistochemical findings corroborate these data, showing increased MBP immunoreactivity and improved myelin organization in the swimming group compared with the injury group.

The combined therapy produced the most balanced neuroprotective profile, restoring myelin thickness, g-ratio values, and SFI scores to near-control levels. We interpret these outcomes as additive rather than synergistic, since both interventions act via complementary mechanisms—dexamethasone mitigating early inflammatory and oxidative cascades [40] and exercise promoting neurotrophic and regenerative pathways [41].

To ensure reproducibility and minimize confounding factors, several methodological refinements were implemented. All swimming sessions were performed during the light phase (10:00–14:00) of a controlled 12:12 h light–dark cycle. This timing was chosen to reduce circadian variability, as locomotor and hormonal responses to exercise can differ between day and night [42]. To control for exercise-induced stress, animals underwent a three-day acclimatization period before surgery and again before the start of the postoperative exercise regimen. Water temperature was strictly maintained at 28 ± 1 °C, a range known to minimize corticosterone elevation while maintaining voluntary swimming behavior [29]. Sessions were continuously observed, and exercise was immediately stopped if signs of fatigue, immobility, or respiratory distress appeared. Daily body weight, grooming, and feeding behavior were monitored to confirm animal welfare. These precautions ensured that the protocol represented moderate, non-aversive physical activity, consistent with recommendations from prior studies employing similar designs [27,28]. The 20 min, three-sessions-per-week protocol was selected because previous reports demonstrated that this duration optimizes neural recovery while preventing overtraining or wound-related complications [21,27]. Shorter sessions (<10 min) may be insufficient to induce neurotrophic adaptations [43], whereas longer or more frequent sessions can increase fatigue or stress levels without additional regenerative benefit [44]. Thus, the chosen regimen represents a moderate-intensity model for postoperative rehabilitation in rodents.

The bioinformatic and molecular docking analyses further substantiate the experimental findings by revealing potential molecular targets and mechanisms through which dexamethasone exerts its neuroprotective effects. The KEGG pathway enrichment analysis identified key signaling cascades associated with inflammation (NF-κB and TNF), oxidative stress (NOS2, NOS3, and PARP1), and metabolic regulation (HIF-1, adipocytokine, and arginine pathways), supporting the observed reduction in oxidative and inflammatory markers in vivo [45,46]. The docking simulations demonstrated strong binding affinities of dexamethasone to NF-κB (≥−6 kcal/mol) and CASP3 (≥−6 kcal/mol), suggesting a direct modulatory influence on inflammatory and apoptotic pathways. Notably, the stable interaction between dexamethasone and Myelin Basic Protein (−8.5 kcal/mol) provides a mechanistic perspective, implying that dexamethasone may also confer myelin stabilization through direct binding to structural myelin components [47,48]. This combined in silico evidence aligns with the immunohistochemical restoration of MBP expression and supports the hypothesis that dexamethasone promotes neuroprotection via both receptor-mediated and structural pathways.

Despite these strengths, certain limitations should be acknowledged. First, only a single dose of dexamethasone (2 mg/kg/day for 10 days) and a single exercise duration were evaluated; therefore, the findings cannot be generalized to other dosing regimens or intensities. Dose–response and time-course studies are needed to identify optimal therapeutic windows. Second, the study relied primarily on MBP immunostaining as a myelin marker; incorporating additional proteins such as myelin protein zero, peripheral myelin protein 22 or neurofilament H would strengthen mechanistic interpretation. Finally, although the local perineural administration of dexamethasone offers translational relevance by limiting systemic exposure, clinical applications will require validation regarding delivery method, safety, and timing relative to rehabilitation onset.

Collectively, our results support the concept that combining an anti-inflammatory pharmacological agent with structured physical exercise can provide additive protection after peripheral nerve injury. The complementary actions of dexamethasone and swimming exercise may help create a favorable microenvironment for axonal regrowth and remyelination, potentially informing multimodal therapeutic strategies for peripheral nerve repair.

## 5. Conclusions

In conclusion, both dexamethasone and swimming exercise independently improved biochemical, histological, and functional outcomes following sciatic nerve crush injury in rats. The combination therapy resulted in the greatest reduction in oxidative stress markers (MDA and MPO), the most pronounced increase in antioxidant enzyme activity (GPX), and near-complete restoration of myelin integrity and MBP expression, as supported by histological, immunohistochemical, and ultrastructural findings. Functional recovery assessed by the SFI also confirmed superior motor performance in the combined group compared with single treatments. These results demonstrate that integrating a local anti-inflammatory corticosteroid with a controlled aerobic exercise protocol produces additive neuroprotective effects that promote axonal preservation and remyelination. Within the tested dose and exercise parameters, this multimodal approach provides measurable structural and functional improvement in peripheral nerve regeneration.

## Figures and Tables

**Table 1:** Biochemical evaluation of oxidative stress markers (MDA, GPx, and MPO) across experimental groups.

Group (*n*:8)	MDA	GPx	MPO	*p*-Value (vs. Injury)
Control	0.19 ± 0.05	115.16 ± 12.48	8.41 ± 1.63	*p* < 0.001
Injury	2.13 ± 0.05	68.1 ± 6.19	25.84 ± 2.47	-
Injury + Swimming	0.98 ± 0.10	94.12 ± 3.72	14.37 ± 2.33	*p* < 0.001
Injury + Dexamethasone	0.67 ± 0.04	81.87 ± 3.99	18.99 ± 1.5	*p* < 0.001
Combination	0.37 ± 0.05	101.91 ± 4.31	10.41 ± 1.97	*p* < 0.001

MDA (nmol/mL): One-way ANOVA: F(4,35) = 1231.46, *p* < 0.001, η^2^ = 0.993. Post hoc (Tukey): All treatment groups showed significantly lower MDA levels compared with the Injury group (*p* < 0.001). GPx (ng/g tissue): One-way ANOVA: F(4,35) = 54.18, *p* < 0.001, η^2^ = 0.861. Post hoc (Tukey): All treatment groups showed significantly higher GPx levels compared with the Injury group (*p* < 0.01). MPO (ng/g tissue): One-way ANOVA: F(4,35) = 96.91, *p* < 0.001, η^2^ = 0.917. Post hoc (Tukey): All treatment groups showed significantly lower MPO levels compared with the Injury group (*p* < 0.001).

**Table 2:** Sciatic Functional Index (SFI) values in experimental groups at postoperative days 1, 7, 14, 21, and 28. Values are expressed as mean ± SD (*n* = 8/group). Higher SFI scores indicate better motor function recovery (0 = normal function, −100 = complete loss). Comparisons across groups and time points were evaluated using two-way repeated measures ANOVA with Tukey’s post hoc test.

Day	Control	Injury	Swimming	Dexamethasone	Combination
1	−5.58 ± 0.52	−86.16 ± 1.66	−85.39 ± 1.35	−85.69 ± 1.11	−85.62 ± 0.96
7	−5.50 ± 0.35	−74.93 ± 2.41	−67.85 ± 0.82	−64.65 ± 2.05	−54.96 ± 2.72
14	−5.50 ± 0.57	−66.31 ± 2.98	−56.07 ± 3.16	−46.65 ± 1.24	−30.53 ± 1.58
21	−5.86 ± 0.39	−57.08 ± 2.97	−41.23 ± 1.73	−34.11 ± 1.21	−14.05 ± 1.98
28	−5.74 ± 0.39	−45.85 ± 2.48	−27.36 ± 0.99	−20.75 ± 1.23	−7.25 ± 0.83

ANOVA (between groups, by time point): Day 1: F(4,35) = 7337.13, *p* < 0.001 (no difference between Combination vs. Swimming or Dex, *p* > 0.05). Day 7: F(4,35) = 1700.51, *p* < 0.001, η^2^ = 0.995. Day 14: F(4,35) = 977.18, *p* < 0.001, η^2^ = 0.991. Day 21: F(4,35) = 986.94, *p* < 0.001, η^2^ = 0.991. Day 28: F(4,35) = 1137.87, *p* < 0.001, η^2^ = 0.992. Selected pairwise comparisons (Tukey, df = 35): Combination vs. Dexamethasone: Day 7 t = 10.16; Day 14 t = 14.96; Day 21 t = 21.54; Day 28 t = 19.60 (all *p* < 0.001). Combination vs. Swimming: Day 7 t = 13.51; Day 14 t = 23.70; Day 21 t = 29.18; Day 28 t = 29.20 (all *p* < 0.001). Combination vs. Injury: Day 7 t = 20.93; Day 14 t = 33.20; Day 21 t = 46.20; Day 28 t = 56.05 (all *p* < 0.001). Day 1: Combination vs. Dex or Swimming *p* > 0.05 (no significant differences).

## Data Availability

The original contributions presented in this study are included in the article/Supplementary Material. Further inquiries can be directed to the corresponding author.

## Supplementary Materials

The following supporting information can be downloaded at https://www.mdpi.com/article/10.3390/antiox14111382/s1. Figure S1. Surgical induction of the sciatic nerve compression model. Figure S2. Representative rat footprint showing measurement points for SFI.
