# Supplementary material for: Evaluation of Dexamethasone and Swimming Exercise as Complementary Interventions in a Rat Sciatic Nerve Injury Model
Source: Antioxidants (Basel). 2025 Nov 20;14(11):1382. doi: 10.3390/antiox14111382 (PMC12649159; doi:10.3390/antiox14111382)
Supplement: Supplementary file 1 [file antioxidants-14-01382-s001.zip › antioxidants-3935463-supplementary.pdf]

Supplementary materials

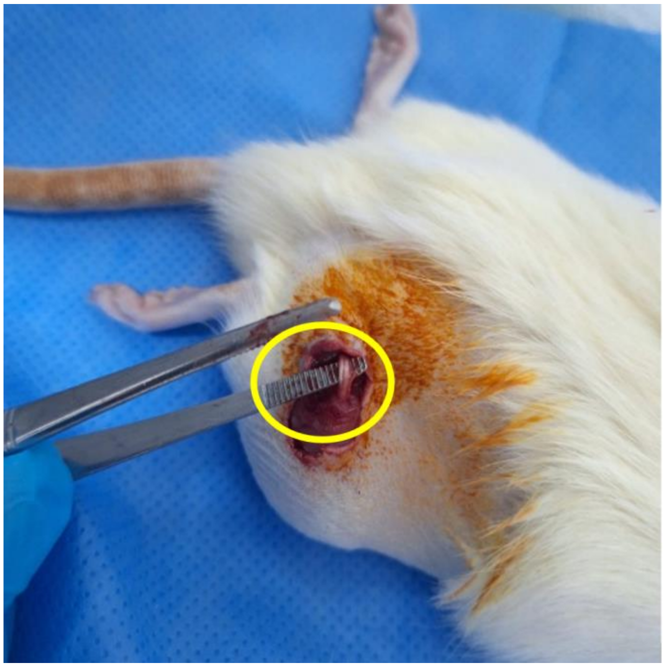

Figure S1. Surgical induction of the sciatic nerve compression model

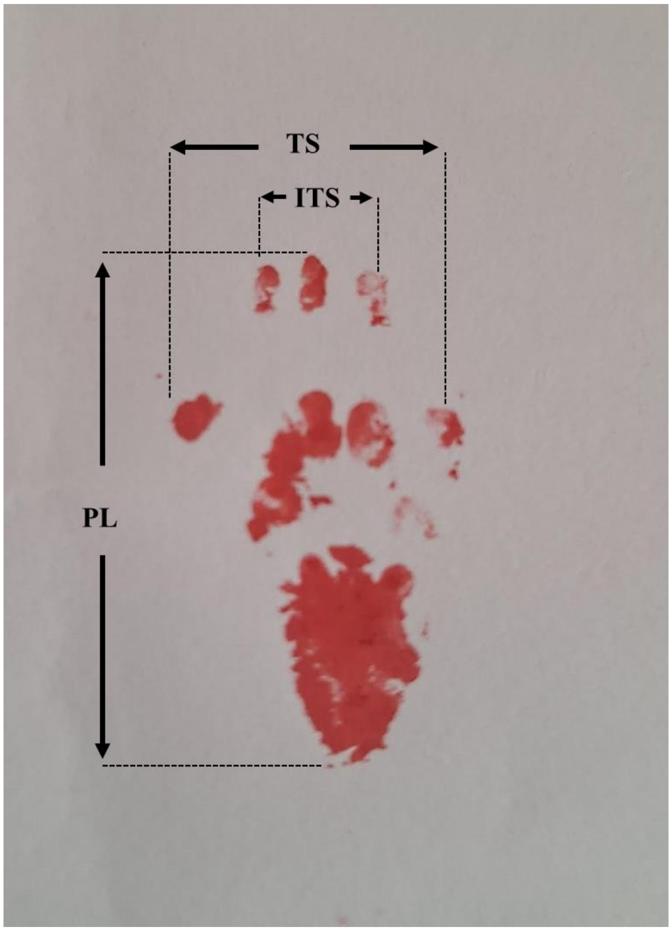

Figure S2. Representative rat footprint showing measurement points for SFI. Paw Length (PL): Distance from the heel to the tip of the third; Toe Spread (TS): Distance between the first and fifth toes; Intermediary Toe Spread (ITS): Distance between the second and fourth toes. Measurements were taken from both the injured (E) and uninjured (N) limbs. SFI was calculated using the following formula: $SFI = -38.3 \times [(EPL - NPL)/NPL] + 109.5 \times [(ETS - NTS)/NTS] + 13.3 \times [(EITS - NITS)/NITS] - 8.8$ .SFI values range from −100 (complete loss of function) to 0 (normal function or full recovery).
